# Supplementary material for: TMT-Based Quantitative Proteomics Analysis Reveals Airborne PM2.5-Induced Pulmonary Fibrosis
Source: Int J Environ Res Public Health. 2018 Dec 31;16(1):98. doi: 10.3390/ijerph16010098 (PMC6339163; doi:10.3390/ijerph16010098)
Supplement: Supplementary file 1 [file ijerph-16-00098-s001.zip › Supplementary fiels/Supplementary table 1.docx]

**Table S1.** The primer sequences for RT-PCR analysis

| Gene | Forward primer (5’-3’) | Reverse primer (5’-3’) |
| --- | --- | --- |
| *LYN* | AGTGCAGGAGCTTTCCTTATCA | CGAGGAGAGATGTAATAGCCACC |
| *MMP9* | GCGTCGTGATCCCCACTTAC | CAGGCCGAATAGGAGCGTC |
| *Collagen I* | TAAGGGTCCCCAATGGTGAGA | GGGTCCCTCGACTCCTACAT |
| *FGF1* | CAGCTCAGTGCGGAAAGTG | TGTCTGCGAGCCGTATAAAAG |
| *CD36* | AGATGACGTGGCAAAGAACAG | CCTTGGCTAGATAACGAACTCTG |
| *GPX2* | AAGCTGCCCTACCCTTATGAT | AAGTTCCAGGACACGTCTGAG |
| *MPO* | GACATGCCCACCGAATGACAA | CAGGCAACCAGCGTACAAAG |
| *COQ6* | GCAGATCGAGTGAAGGTTCTC | GTCTGGAGGGTACTGCCATCA |
| *CYP2A5* | TGGTCCTGTATTCACCATCTACC | ACTACGCCATAGCCTTTGAAAA |
| *TGF-β* | CTTCAATACGTCAGACATTCGGG | GTAACGCCAGGAATTGTTGCTA |
| *β-actin* | GTGACGTTGACATCCGTAAAGA | GCCGGACTCATCGTACTCC |
